# Stabilizing the Proteomes of Acute Myeloid Leukemia Cells: Implications for Cancer Proteomics

**DOI:** 10.1016/j.mcpro.2024.100716

**Published:** 2024-01-12

**Authors:** Robert W. Sprung, Qiang Zhang, Michael H. Kramer, Matthew C. Christopher, Petra Erdmann-Gilmore, Yiling Mi, James P. Malone, Timothy J. Ley, R. Reid Townsend

**Affiliations:** 1 Division of Endocrinology, Metabolism, and Lipid Research, Department of Internal Medicine, Washington University School of Medicine, St. Louis, Missouri, USA; 2 Division of Oncology, Department of Internal Medicine, Washington University School of Medicine, St. Louis, Missouri, USA

**Keywords:** acute myeloid leukemia, DFP, protease inhibition, nontryptic peptides, isobaric labeling proteomics, label-free proteomics

## Abstract

Previous work has shown that inhibition of abundant myeloid azurophil granule-associated serine proteases (ELANE [neutrophil elastase], PRTN3 [protease 3], and CTSG [Cathepsin G]) is required to stabilize some proteins in myeloid cells. We therefore hypothesized that effective inhibition of these proteases may be necessary for quantitative proteomic analysis of samples containing myeloid cells. To test this hypothesis, we thawed viably preserved acute myeloid leukemia cells from cryovials in the presence or the absence of diisopropyl fluorophosphate (DFP), a cell-permeable and irreversible serine protease inhibitor. Global proteomic analysis was performed, using label-free and isobaric peptide-labeling quantitation. The presence of DFP resulted in an increase of tryptic peptides (14–57%) and proteins (9–31%). In the absence of DFP, 11 to 31% of peptide intensity came from nontryptic peptides; 52 to 75% had cleavage specificity consistent with activities of ELANE–PRTN3. Treatment with DFP reduced the intensity of nontryptic peptides to 4-8% of the total. ELANE inhibition was 95%, based on diisopropyl phosphate modification of active site serine residue. Overall, the relative abundance of 20% of proteins was significantly altered by DFP treatment. These results suggest that active myeloid serine proteases, released during sample processing, can skew quantitative proteomic measurements. Finally, significant ELANE activity was also detected in Clinical Proteomics Tumor Analysis Consortium datasets of solid tumors (many of which have known myeloid infiltration). In the pancreatic cancer dataset, the median percentage of nontryptic intensity detected across patient samples was 34%, with many patient samples having more than half of their detected peptide intensity from nontryptic cleavage events consistent with ELANE-PRTN3 cleavage specificity. Our study suggests that *in vitro* cleavage of proteins by myeloid serine proteases may be relevant for proteomic studies of any tumor that contains infiltrating myeloid cells.

## Highlights

• Acute myeloid leukemia cells contain abundant endogenous serine proteases.
• These endogenous proteases efficiently generate nontryptic peptides.
• The generation of nontryptic peptides alters proteomic quantitation.
• DFP effectively minimized generation of nontryptic peptides.

## Graphical Abstract

**Figure:**
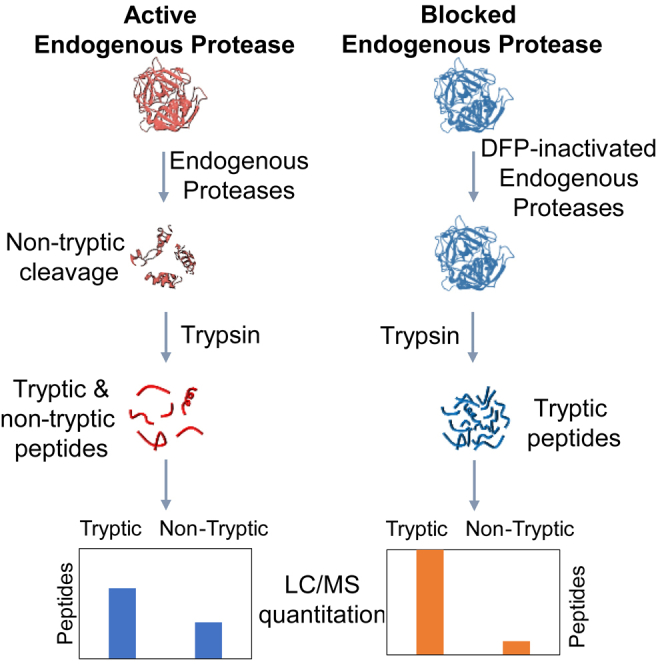

## In Brief

Effective inhibition of endogenous serine proteases is essential for accurate protein quantitation using tryptic peptides. Diisopropyl fluorophosphate is a membrane-permeable and irreversible serine protease inhibitor. Inclusion of diisopropyl fluorophosphate prior to lysis of acute myeloid leukemia cells resulted in a large increase in identification of tryptic peptides (∼36%) and proteins (∼20%). Nontryptic peptides produced by myeloid serine proteases were also detected in Clinical Proteomics Tumor Analysis Consortium data for solid tumor samples, suggesting broad relevance for these findings.

Quantitative proteomics depends on the reproducible detection of tryptic peptides from complex mixtures of proteins, using high-resolution LC–MS. Of the many peptides generated for any given protein by trypsin digestion, only a few are consistently identified for quantitation of these prototypic peptides (1). Peptide preparation processes that affect the reproducible generation of prototypic peptides may result in inaccurate protein quantitation; endogenous proteases present in some cell types may therefore alter proteomic results. For this reason, low temperature, protein denaturation, and protease inhibitors are routinely employed during cell lysis to minimize the activity of endogenous proteases (2).

Although the protease content of cells varies widely, there have been few studies to address the effectiveness of standard protease inhibitor approaches to minimize endogenous protease activity. Specifically, cells of the myeloid lineage (both neutrophils and monocytes) contain large quantities of four coregulated serine proteases (ELANE [neutrophil elastase], PRTN3 [protease 3] and CTSG [Cathepsin G]) that are normally stored as inert molecules in azurophilic granules, a specialized lysosome (3, 4). Indeed, previous studies have suggested that increased quantities of protease inhibitors are required for quantitative proteomic (3, 4) and phosphoproteomic studies of neutrophils (5). Our studies have shown that inhibition of ELANE was required to detect the full-length PML::RARA fusion protein in acute promyelocytic leukemia cells (6). In this study, we posited that inhibition of endogenous myeloid proteases with a membrane-permeable serine protease inhibitor (diisopropyl fluorophosphate [DFP]) may improve global proteomic analysis of acute myeloid leukemia (AML) cells that had previously been viably cryopreserved (7, 8). We compared the protease inhibition protocol used by the Clinical Proteome Tumor Analysis Consortium (CPTAC) (2) with a modified method that included DFP addition during the cell thawing process, prior to cell lysis. In the absence of treatment with DFP, we found that quantitation of ∼20 to 38% of cell proteins was significantly (*p* < 0.05) altered in cryopreserved AML cells, as measured by tandem mass tag (TMT) and label-free quantitation (LFQ) strategies, respectively. We used the validated proteomic method that had been adopted by CPTAC for tumors to prepare peptides (9) for label-free and isobaric-labeling quantitation. Analysis of the terminal residues of nontryptic peptides revealed that ELANE and PRTN3 accounted for most (52–75%) of the endogenous protease activity in AML cells not treated with DFP. Moreover, we found that DFP efficiently inhibited ELANE, with over 95% of the signal for the active site serine peptide being DFP-adduct modified in DFP-treated samples. We also found that in the absence of DFP treatment prior to cell lysis, a marked increase in the number of nontryptic peptides was detected. Finally, since several recent studies have suggested that tumor-associated neutrophils can influence the outcomes of patients with solid tumors (10, 11, 12), we evaluated CPTAC datasets for pancreatic (13) and squamous cell lung cancer (14) (both associated with neutrophil infiltration) for evidence of ELANE activity in these sample sets. Indeed, the unique cleavage specificities of ELANE and PRTN3 (and also the proteases themselves) were detected in many samples. This approach can be used to accurately detect the presence of myeloid cells in solid tumor proteomic studies, which may be relevant for accurate proteomic analysis.

## Experimental Procedures

### Preparation of Cell Lysates

Cell lysates were prepared from freshly banked AML bone marrow cells (from buffy coat preparations) from presentation samples, which were viably cryopreserved, as previously described (15). All human samples were collected with approval from the Washington University Institutional Review Board (approved banking protocol #201011766) in accordance with the Declaration of Helsinki principles. Cells were thawed in the presence or the absence of DFP (Sigma; catalog no.: D0879-1G). The handling and disposal of DFP was performed under the guidelines from the Washington University Environmental Health and Safety Office. Prior to the thawing of cells, a waste beaker with 2 N sodium hydroxide (NaOH; Fisher BioReagents; catalog no.: BP359-500) was prepared for discarded pipette tips and other plastic that would come in contact with DFP. A 2 l beaker with 500 ml of NaOH was found to be sufficient for eight samples. The beaker and waste remained in the fume hood for 48 h, then the plastic waste was bagged, and the liquid waste was decanted into a bottle for collection by our Environmental Health Service.

Cryovials in sets of two were removed from liquid nitrogen. The vials were warmed briefly in a 37 °C water bath until the frost was off the outside of the tube, without thawing the contents of the vial. Fetal bovine serum (0.75 ml) at room temperature, with or without DFP, was added to each vial containing ∼1 ml of cell suspension. The final concentration of DFP was 2 mM. The fetal bovine serum ± DFP was added to the partially thawed contents of the cryovial, which was then transferred to a new 5 ml tube. Cells were kept on ice for 15 min and then centrifuged at 1200 RPM at 4 °C for 5 min. The supernatants were discarded into the beaker with NaOH. Cell pellets were resuspended in 1 ml PBS, followed by an additional centrifugation, resuspension, and centrifugation, discarding all waste into the beaker with 2 N NaOH to inactivate any residual DFP. This wash process was repeated once.

We previously determined that the yield of protein from fresh nonfrozen AML cells is on the order of 300 μg per 10,000,000 cells. As recently demonstrated (15), we were able to obtain sufficient material for deep scale proteomics and phosphoproteomics from 44 banked human AML samples. Our banking protocol attempts to viably freeze 10 million cells per cryovial, but there is variation from sample to sample and day to day that reflects sample heterogeneity, and technical issues associated with time of day and sample transport. Specifically, we started by thawing one cryovial per case, and that was all that was needed for 22 of the 44 successful samples (15). About 19 samples required two cryovials to obtain adequate protein, and three of them required three cryovials. Nine samples did not have adequate yields. The average numbers of cells used per successful case was 15.8 million (range = 6.8 to 29.6 M). The average protein yield per successful sample was 483 μg, with a range of 275 to 1408 μg for the combined deep scale (TMT and LFQ) and phosphoproteomic analyses. All 44 successful samples had a starting material threshold of 275 μg. For the nine unsuccessful samples, all were below that threshold, with an average yield of 92 μg.

### Preparation of Protein Lysates

The cell pellets were solubilized in 100 μl of Tris urea lysis buffer recommended by CPTAC (2) (50 mM Tris–HCl, pH 8.0, containing 8 M urea (Sigma; catalog no.: U4884-500g), 75 mM sodium chloride (Sigma–Aldrich; catalog no.: 71376), 1 mM EDTA (Sigma–Aldrich; catalog no.: E1644-100G), 10 mM sodium fluoride (Sigma–Aldrich; catalog no.: S7920), phosphatase inhibitor cocktail 2 (Sigma–Aldrich; catalog no.: P5726) (1:100 dilution), phosphatase inhibitor cocktail 3 (Sigma–Aldrich; catalog no.: P0044) (1:100 dilution), 2 μg/ml aprotinin (Sigma–Aldrich; catalog no.: A6103), 10 μg/ml leupeptin (Roche; catalog no.: 11017101001), and 1 mM PMSF (Sigma–Aldrich; catalog no.: 78830, pH 8.0).

The samples were transferred with a single lysis buffer rinse (50 μl) to Covaris milliTUBEs (Covaris; catalog no.: 520135) with adaptive focus acoustics fiber for focused ultrasonication. The lysates were sonicated (Covaris model S220X) for 12 min (peak incident power: 70 W, duty factor: 50%, cycles/burst: 200, time: 12 min, and temperature: 5–8 °C), placed on ice, and transferred to 1.7 ml tubes (Axygen; catalog no.: NCT-175-C). The lysates were spun at 16,000*g* in a microcentrifuge (Eppendorf model 5424) for 30 min at 4 °C.

The supernatants were collected, and protein concentration was determined using a Pierce BCA Protein Assay Kit (Pierce; catalog no.: 23225). The lysates were aliquoted (15 μg) into 0.5 ml tubes and stored at −80 °C for the preparation of peptides, as previously described (2), with minor modifications. A reference pool was prepared by pooling aliquots of each lysate prior to digestion (Supplemental Table S1). Proteins were reduced with 6 mM DTT (Pierce; catalog no.: 20291) for an hour at 37 °C, followed by alkylation with 13 mM iodoacetamide (Pierce; catalog no.: A39271) for 45 min at room temperature in the dark.

Peptides were prepared after sequential digestion with endoprotease Lys C (Wako Chemicals; catalog no.: 129-02541) (1 mAU) and trypsin (Promega; catalog no.: V5113) (1 μg).

The peptides were acidified to 1% (v/v) TFA (Sigma; catalog no.: 91707) and desalted using two microtips (porous graphite carbon; BIOMEKNT3CAR) (Glygen) on a Beckman robot (Biomek NX), as previously described (16). The peptides were eluted with 60 μl of 60% (v/v) acetonitrile (MeCN; J.T. Baker; catalog no.: 9829-03) in 0.1% (v/v) TFA and dried in a Speed-Vac (Thermo Scientific; model no.: Savant DNA 120 concentrator). The peptides were dissolved in 20 μl of 1% (v/v) MeCN in water. An aliquot (10%) was removed for quantitation using a Quantitative Fluorometric Peptide Assay kit (Pierce; catalog no.: 23290). An aliquot (1 μg) was removed and transferred to autosampler vials (Sun-Sri; catalog no.: 200046) for LFQ. Peptides were dried and stored at −80 °C. Another aliquot (1 μg) of the samples and two identical reference pools were transferred into 1.7 ml Eppendorf tubes for subsequent TMT labeling (Supplemental Table S1). Peptides were lyophilized and stored at −80 °C.

The lyophilized peptides were dissolved in 40 μl of Hepes buffer (Alfa Aesar; catalog no.: J63218) (100 mM, pH 8.5) and labeled using the TMT-11 reagent kit (Thermo Fisher Scientific; catalog no.: A34808), following the vendor protocol but increasing the TMT reagent to peptide ratio to 32:1. The efficiency of labeling was >98% by LC–MS.

The labeled samples and two reference pools were combined, dried, and dissolved in 120 μl of 1% (v/v) formic acid (FA; Sigma–Aldrich; catalog no.: 56302). The TMT-11-labeled samples were desalted as described previously for the unlabeled peptides. TMT-labeled peptides from desalting were collected into the autosampler vials (Sun-Sri; catalog no.: 200046), dried, and stored at −80 °C.

### uPLC Orbitrap-MS

The TMT-labeled and LFQ samples were analyzed using ultra–high performance mass spectrometry (MS) (17) using a hybrid quadrupole Orbitrap LC–MS System, Q-Exactive PLUS interfaced to an EASY-nano-LC 1000. A 75 μm i.d. ×50 cm Acclaim PepMap 100 C18 RSLC column (Thermo Scientific) was equilibrated with 100% solvent A (1% FA) on the nano-LC for a total of 11 μl at 700 bar pressure. Samples in 1% FA (v/v) were loaded at a constant pressure of 700 bar. For TMT-labeled samples, peptide chromatography was initiated with mobile phase A (1% FA) containing 5% solvent B (100% MeCN, 1% FA) for 1 min, then increased to 25% B over 195 min, to 35% B over 40 min, to 70% B over 6 min, to 95% B over 2 min and held at 95% B for 18 min, with a flow rate of 250 nl/min.

For LFQ samples, peptide chromatography was initiated with mobile phase A (1% FA) containing 2% solvent B (100% MeCN, 1% FA) for 5 min, then increased to 20% B over 100 min, to 32% B over 20 min, to 95% B over 1 min, and held at 95% B for 19 min, with a flow rate of 250 nl/min.

Data were acquired in data-dependent mode. Full-scan mass spectra were acquired with the Orbitrap mass analyzer using a scan range of *m/z* = 375 to 1500 and a mass resolving power set to 70,000. Twelve data-dependent high-energy collisional dissociations were performed with a mass resolving power at 35,000, a fixed lower value of *m/z* 100, an isolation width of 1.2 Da, and a normalized collision energy setting of 32. The maximum injection time was 60 ms for parent-ion analysis and 120 ms for product-ion analysis. Ions that were selected for MS–MS were dynamically excluded for 40 s. The automatic gain control was set at a target value of 3e6 ions for full MS scans and 1e5 ions for MS2.

### Peaks Searches

Data from the mass spectrometer were analyzed using Peaks software with the default settings for peak list generation (18) (Bioinformatics Solutions; Peaks Studio, version 10.6). Peaks was set up to search against a UniProt reference database of human proteins (downloaded January 2018, 21,005 entries). For digestion characteristics, enzyme was set to “none” and digestion mode was set to “unspecific,” allowing cleavage after any amino acid, and the default setting of 100 missed cleavages was considered. Searches were performed with a fragment ion mass tolerance of 20 ppm and a parent ion tolerance of 20 ppm. For all searches, carbamidomethylation of cysteine was specified in Peaks as a fixed modification. Deamidation of asparagine, deamidation of glutamine, formation of pyroglutamic acid from N-terminal glutamine, acetylation of protein N terminus, and oxidation of methionine were specified as variable modifications. Peptides were exported at a 1% false discovery rate based on a decoy database. Peptides with a Peaks feature area of zero were considered not observed.

### TMT Identification of Proteins

Data from the mass spectrometer were converted to peak lists using Proteome Discoverer (version 2.1.0.81; Thermo Fisher Scientific). The MS2 spectra with charges +2, +3, and +4 were analyzed using Mascot software (19) (version 2.7.0; Matrix Science). The searches were performed with a fragment ion mass tolerance of 0.02 Da and a parent ion tolerance of 20 ppm. MS–MS spectra from the AML dataset were set up to search against a RefSeq (version July 2018) database of human proteins (41,850 entries) and common contaminant proteins (cRAP, version 1.0, January 2012; 116 entries). MS–MS spectra from the pancreatic (13), lung carcinoma (14), and breast cancer (20) datasets were downloaded from the CPTAC data portal (https://proteomic.datacommons.cancer.gov/pdc/) and set up to search against a UniProt database of human proteins (20,512 entries, version October 2020) and common contaminant proteins (cRAP, version 1.0, January 1, 2012; 116 entries). Searches were performed at either no enzymatic specificity or assuming the digestion enzyme of trypsin/P with a maximum of four missed cleavages allowed. Carbamidomethylation of cysteine was specified as a fixed modification. Deamidation of asparagine, formation of pyroglutamic acid from N-terminal glutamine, acetylation of protein N terminus, and oxidation of methionine were specified as variable modifications with searches at trypsin/P enzymatic specificity. Acetylation of protein N terminus and oxidation of methionine were specified as variable modifications with searches at no enzymatic specificity. Peptide-spectrum matches (PSMs) were filtered at 1% false discovery rate by searching against a reversed database, and the ascribed peptide and protein identities were accepted.

### TMT Protein Relative Quantitation

The processing, quality assurance, and analysis of TMT data were performed with proteoQ (https://github.com/qzhang503/proteoQ), a tool developed with the tidyverse approach (21). For tidyverse: one can “Easily install and load the 'Tidyverse' R package” (22) under the free software environment for statistical computing and graphics, R (23) and RStudio (24).

For normalization of TMT data, reporter-ion intensities under 11-plex TMT channels were converted to logarithmic ratios at base 2, relative to the average reporter-ion intensity of reference samples within an 11-plex TMT.

Within each sample, Dixon’s outlier removals were carried out recursively for peptides with greater than two identifying PSMs. The median of the ratios of PSM that can be assigned to the same peptide was first taken to represent the ratios of the incumbent peptide. The median of the ratios of peptides was then taken to represent the ratios of the incumbent protein.

To align protein ratios across samples, likelihood functions were first estimated for the log ratios of proteins using finite mixture modeling, assuming two-component Gaussian mixtures (25). The ratio distributions were then aligned in that the maximum likelihood of the log ratios is centered at zero for each sample. Scaling normalization was performed to standardize the log ratios of proteins across samples. To discount the influence of outliers from either log ratios or reporter-ion intensities, the values between the fifth and 95th percentile of log ratios and fifth and 95th percentile of intensity were used in the calculations of the standard deviations.

### Experimental Design and Statistical Rationale

Presentation bone marrow samples from five individuals with AML were analyzed with LFQ and TMTs. Proteomic process replicates were performed in triplicate for both DFP-treated and untreated AML samples. Statistical comparisons were made between cells treated and untreated with DFP. AML samples were randomized across TMT plexes and channels.

For LFQ data with replicate analyses, a *t* test was used to determine significant differences.

Metric multidimensional scaling and principal component analysis of protein log2 ratios were performed with the base R function stats::cmdscale and stats:prcomp, respectively. Heat-map visualization of protein log2 ratios was performed with pheatmap (https://CRAN.R-project.org/package=pheatmap). Linear modelings were performed using the contrast fit approach in limma (26) to assess the statistical significance in protein abundance differences between indicated groups of contrasts. Adjustments of *p* values for multiple comparison were performed with Benjamini–Hochberg correction (27).

## Results

### Inhibition of Neutrophil Serine Proteases Prevents Cleavage of Proteins During Sample Processing

**Fig. 1:**
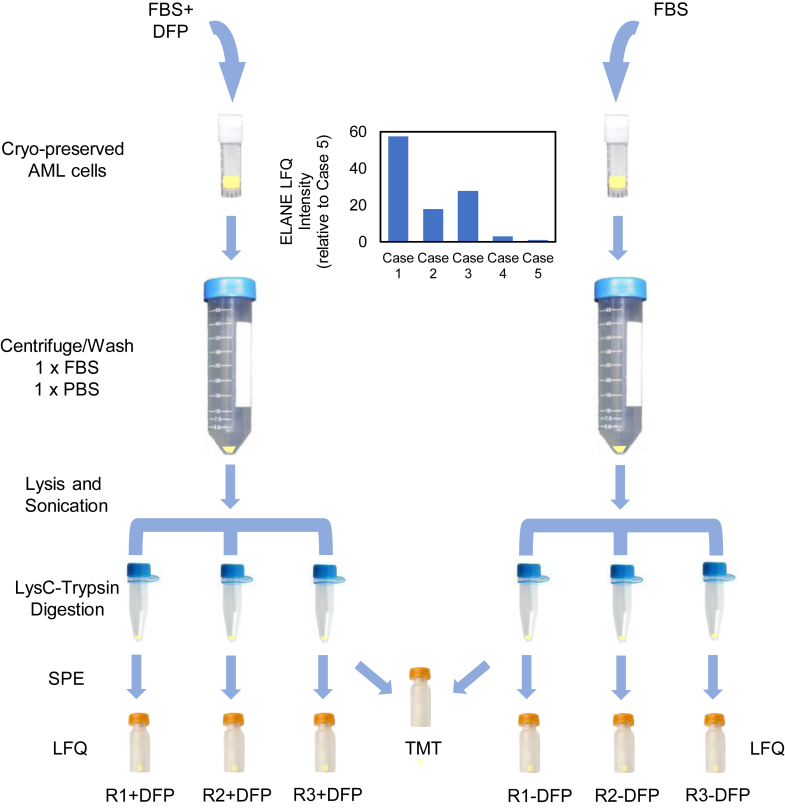
**Proteomic workflows for the preparation of acute myeloid leukemia (AML) cells from banked samples.** Label-free quantitation (LFQ) and tandem mass tagging (TMT) protocols are detailed in the “Experimental procedures” section. The *inset* shows ELANE LFQ intensity (relative to case 5) in cases 1 through 5. ELANE, neutrophil elastase.

**Fig. 2:**
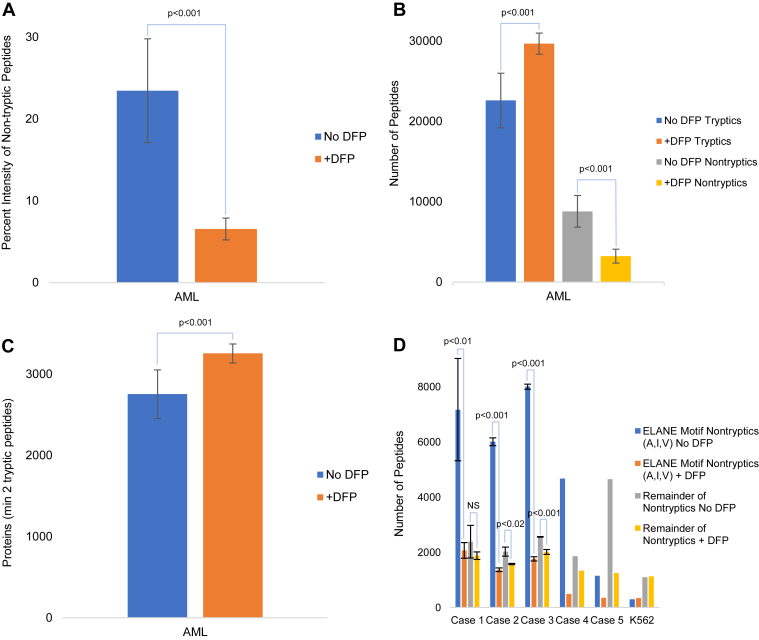
**Effect of DFP on observed proteins and peptides from AML.***A*, grouped bar chart showing the percent intensity for nontryptic peptides identified in the presence or the absence of DFP during proteomic analysis of five AML cases (Supplemental Tables S1 and S2). Error bars represent standard deviation of 11 replicate digests, and T-statistic *p* values are shown. *B*, grouped bar chart of peptides identified in the presence and absence of DFP (error bars represented as in *A*). *C*, grouped bar chart of proteins identified by two or more tryptic peptides (error bars represented as in *A*). *D*, grouped bar chart of nontryptic peptides classified by whether they have ELANE–PRTN3 cleavage specificity. Error bars represent standard deviation of triplicate digests for cases 1 to 3, and T-statistic *p* values for these cases are shown; NS indicates no significant difference. AML, acute myeloid leukemia; DFP, diisopropyl fluorophosphate; ELANE, neutrophil elastase; PRTN3, protease 3.

We selected five AML cases with high, intermediate, or low expression of one of the major neutrophil serine proteases, ELANE (Fig. 1 and Supplemental Table S1) and, as a negative control, a human leukemia cell line (K562) that does not express ELANE. We utilized the CPTAC processing method (2) with and without DFP treatment prior to cell lysis (Fig. 1). The urea lysis buffer contained the protease inhibitors aprotinin, PMSF, and leupeptin. After acquiring data on mass spectrometers, we determined the number of tryptic and nontryptic peptides identified using a database search allowing for cleavage at any amino acid residue (Fig. 2, Supplemental Table S2). We observed an average of 23.5% nontryptic peptide intensity relative to total peptide intensity for AML cases without DFP and 6.6% intensity in the presence of DFP (Fig. 2*A*). Strikingly, samples processed using standard methods without DFP contained a large fraction of nontryptic peptides (an average of 8792 nontryptic peptides, representing 28% of observed peptides; Fig. 2*B*). Importantly, these peptides would be undetected in a standard proteomics database search used to quantify proteins. As expected, K562 cells (which express very small amounts of the myeloid serine proteases) demonstrated many fewer nontryptic peptides (1382; 5% of observed peptides).

When DFP was added to the cryopreserved pellet prior to cell lysis, there was a marked reduction (63.5%) in nontryptic peptides identified in the AML samples, but not in K562 cells, as expected (Fig. 2*B*, Supplemental Table S2). Furthermore, the presence of DFP during cell preparation resulted in a 31.4% increase in the number of identified tryptic peptides in AML cases (average increase of 7100 tryptic peptides; Fig. 2*B*). These results demonstrate that DFP addition prior to cell lysis results in a marked increase in the number of identified tryptic peptides (Fig. 2*B* and Supplemental Table S3). When DFP was present, there was a concomitant 18.2% increase in the numbers of proteins identified by at least two tryptic peptides for AML samples (Fig. 2*C* and Supplemental Table S4).

To assess whether these nontryptic peptides were in fact caused by myeloid serine protease activity, and to determine which of the proteases (ELANE, CTSG, PRTN3, and NSP4) was responsible, we examined the C-terminal and flanking N-terminal amino acid residues of the nontryptic peptides to determine cleavage specificity (Fig. 2*D* and Supplemental Fig. S1). ELANE and PRTN3 have strict cleavage specificity, cleaving proteins C-terminal to alanine [Ala], isoleucine [Iso], and valine [Val] residues (but not leucine), whereas CTSG cleaves preferentially at tryptophan, leucine, phenylalanine, and tyrosine residues (28).

For the high ELANE-expressing samples (cases 1, 2, and 3), cleavage after Ala, Iso, and Val residues was observed in 75 to 78% of the nontryptic peptides in the absence of DFP, suggesting that the activity of ELANE or PRTN3 is responsible for generating these peptides (Fig. 2*D*). Strikingly, the addition of DFP greatly diminished only those peptides consistent with ELANE–PRTN3 cleavage (Ala [A], Iso [I], and Val [V]) but not the remainder of nontryptic peptides identified (Fig. 2*D*). This strongly suggests that the primary effect of DFP treatment is to reduce the generation of nontryptic peptides through the inhibition of ELANE–PRTN3.

Among the AML cases tested, case 5 demonstrated a unique nontryptic cleavage specificity, with cleavage at leucine and phenylalanine being the most frequent nontryptic cleavage events in the absence of DFP (Supplemental Fig. S1). This stands in contrast to case 1, where the most frequent nontryptic cleavage events occurred at Ala, Iso, and Val, consistent with ELANE–PRTN3 activity. Despite the unique nontryptic cleavage specificity observed in case 5 (most consistent with CTSG activity), DFP markedly reduced generation of these peptides, since it is also a potent inhibitor of CTSG (Fig. 2*D*).

### DFP modification of Proteins is Highly Specific for Serine Proteases

DFP is a highly reactive organophosphate compound that may lead to modification of proteins other than enzymes with active site serines. We next explored whether the DFP effect we observed might be due to protein destruction. We performed a database search allowing for a mass shift of +164.06, corresponding to the additional weight of the DFP adduct (29) on the hydroxyl amino acids serine, threonine, and tyrosine. The search identified a total of 56,849 tryptic peptides from 5358 proteins with a minimum of two unique tryptic peptides (Supplemental Table S5). However, only 32 DFP modification sites with an A-score >20 were identified. Furthermore, we found that 80% of the observed DFP-adduct modified peptide intensity was derived from active site serine modification of ELANE (QAGVCFGDSGSPLVCNGLIHGIASFVR) and CTSG (GDSGGPLLCNNVAHGIVSYGK). These sequences were found predominantly in the modified forms, with 90 to 95% of the measured intensity of the active site serine peptides being DFP modified. These results show that the DFP adduct modification is highly specific, primarily reacting with active site serine. Defining the proportion of modified active site serine peptides serves as a measure of the efficiency of inhibition of serine proteases and proves that DFP “hits its target.” Accordingly, DFP treatment had a minor effect on the integrity of the proteome.

### Inhibition of Neutrophil Serine Proteases Enables Accurate Protein Quantitation

We next defined the effect of DFP treatment on the global quantitative proteomic assessments of AML cells. We followed the CPTAC deep-scale protocol (2) that has been used in multiple large-scale studies of solid tumors and leukemia.

**Fig. 3:**
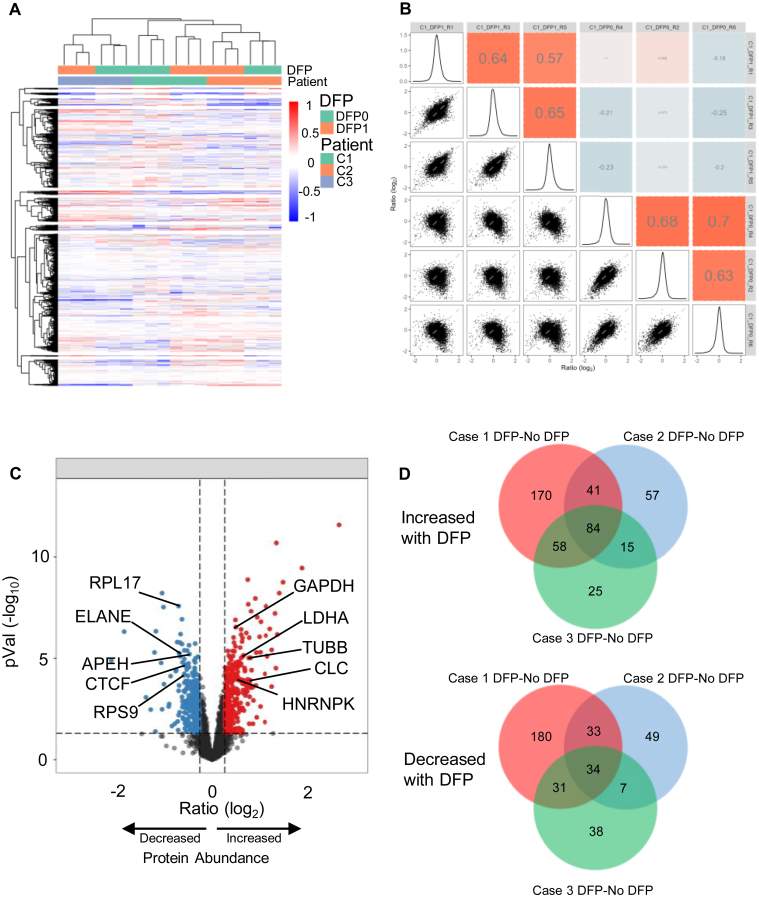
**Proteomic analysis (TMT-labeled tryptic peptides) of AML cases with high expression of ELANE in the presence or the absence of DFP.***A*, uunsupervised protein hierarchical cluster analysis for three cases, each with six LysC/trypsin digest replicates (3 +DFP and three no DFP). Supplemental Table S5 contains a complete listing of proteins represented in this analysis. *B*, pairwise correlations of the peptide relative abundances for the six replicates of case 1, three in the presence and three in the absence of DFP. *C*, relative fold change and *p* values generated from digestion replicate determinations of protein abundance for AML case 1, comparing those treated with DFP to those without DFP. Increased abundance indicates more protein measured in the replicates treated with DFP. *D*, Boolean analysis of proteins that changed significantly (*p* < 0.05) with DFP treatment in each of the three cases. Proteins increasing with DFP treatment are shown in the *upper* Venn diagram, and those decreasing with DFP treatment are shown in the *lower* Venn diagram. A selection of the shared proteins is shown in *C*. AML, acute myeloid leukemia; DFP, diisopropyl fluorophosphate; ELANE, neutrophil elastase; TMT, tandem mass tag.

The effect of DFP treatment on the relative abundances of AML cell proteins is shown as an unsupervised protein heat map of 4216 proteins detected in the three AML high ELANE-expressing cases (cases 1, 2, and 3) performed with six process replicates, three with and three without DFP (Fig. 3, panel *A*, Supplemental Table S6; relative abundances of labeled peptides are provided in Supplemental Table S7). In this analysis, we observed excellent reproducibility among the process replicates, as is apparent from the pattern similarities within each case. These samples cluster by patient and DFP treatment, since each case forms a cluster with clear separation of the DFP-treated and DFP-untreated samples.

Process reproducibility was also high. The average Pearson's correlation for the DFP-treated samples was for case 1 (*r* = 0.62; Fig. 3*B*), case 2 (*r* = 0.77), and case 3 (*r* = 0.83). For the untreated samples, the average pair-wise Pearson's correlations were for case 1 (*r* = 0.67; Fig. 3*B*), case 2 (*r* = 0.74), and case 3 (*r* = 0.84). The protein abundances between the treated and untreated samples showed a poor correlation or a negative correlation (*r* = −0.25–0.088). These results highlight the dramatic effect of DFP treatment on the quantitative proteomic analysis of AML samples. This marked DFP effect was also observed by label-free proteomic analysis of tryptic peptides from the same AML case (Supplemental Fig. S2).

The proteins detected from case 1 that were significantly affected by the presence of DFP (*p* < 0.05) are shown in the volcano plot (Fig. 3*C*), with 353 proteins had higher abundance with DFP treatment (including GAPDH, lactate dehydrogenase A [LDHA], CLC, HNRNPK, and TUBB) and 278 proteins had lower abundance (including RPL17, ELANE, CTCF, RPS9, and APEH [acylpeptide hydrolase]). Among the three AML cases, the total number of significantly increased proteins was 450, and the number of significantly decreased proteins was 372 (Supplemental Table S8). Similarly, from the LFQ analysis of AML case 1, 4684 proteins were identified (Supplemental Table S9) and 38% of the proteome was found to be significantly changed with DFP treatment (883 increased and 882 decreased, Supplemental Table S10).

Across the three AML cases, there were 84 common proteins increased with DFP treatment and 34 common proteins decreased with DFP treatment (Fig. 3*D*; Supplemental Table S8). About 105 proteins showed increased abundance in at least two of the three AML cases analyzed, and 198 proteins showed decreased abundance. Therefore, ∼20% of the quantified proteome is found to significantly change in response to DFP treatment. While many of the altered proteins were found to be consistently changed across the three AML cases, there is also significant variability across cases, perhaps reflecting genetic and proteomic heterogeneity of primary AML samples.

To further investigate the basis of the altered quantitation, we analyzed the abundances of tryptic and nontryptic peptides in the context of sequence coverage of shared proteins affected in the high ELANE-expressing AML cases (Supplemental Table S8).

**Fig. 4:**
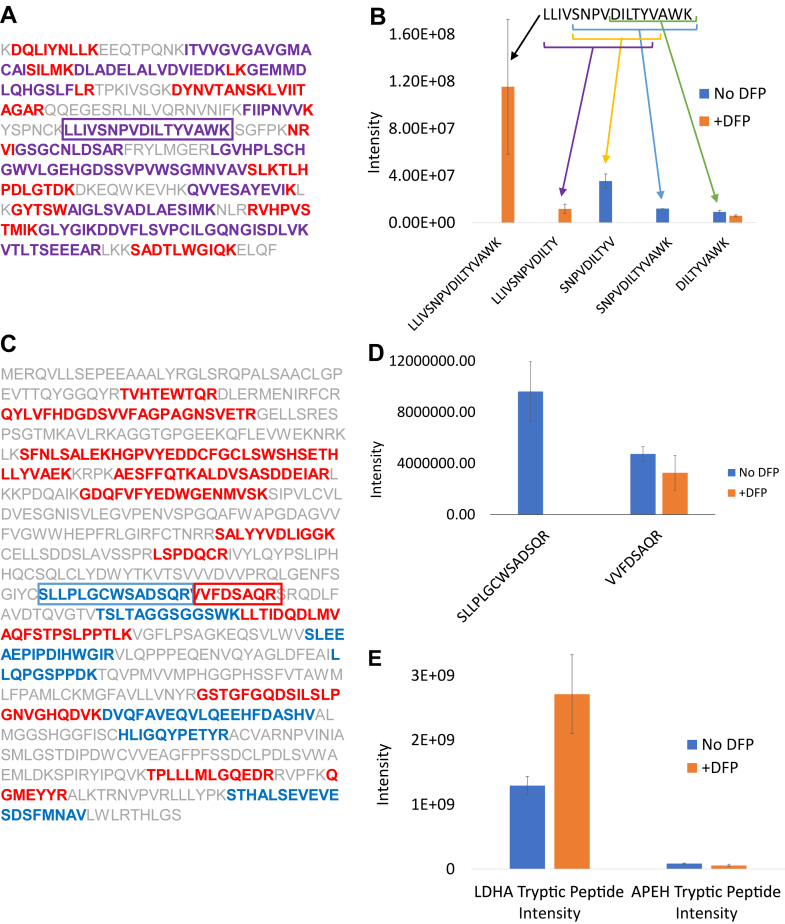
**Sequence coverage and tryptic and nontryptic peptide intensities for proteins that increase or decrease with DFP treatment.***A*, peptides observed with and without DFP (lactate dehydrogenase A [LDHA]—case 1). Tryptic peptides are shown in *red*, nontryptic peptides in *blue*, and peptide sequences common to both appear in *purple*. *B*, tryptic and nontryptic peptide intensities for sequences indicated in *A* (Supplemental Table S4). *C*, peptides observed with and without DFP (APEH–case 1). Tryptic peptides are shown in *red* and nontryptic peptides in *blue*. *D*, tryptic and nontryptic peptide intensities for sequences indicated in *C* (Supplemental Table S4). *E*, summed tryptic peptide intensities from LFQ data for LDHA and APEH in the presence and absence of DFP. Error bars represent standard deviation for triplicate digests. APEH, acylpeptide hydrolase; LDHA, lactate dehydrogenase A; LFQ, label-free quantitation.

The sequence coverage of the 21 tryptic and 34 nontryptic peptides for LDHA revealed that all the identified nontryptic peptide sequences were contained within the observed tryptic sequences (Fig. 4*A*; Supplemental Table S2). Figure 4*B* shows the change in intensity for a selected LDHA tryptic peptide (enclosed by *box* in Fig. 4*A*) and its nontryptic subsequences. DFP was required to observe this tryptic peptide and markedly suppressed the detection of the nontryptic subsequences. This example demonstrates how DFP can limit the generation of nontryptic peptides, enabling the complete tryptic signal to be measured.

Unexpectedly, many proteins showed a decrease in apparent abundance with DFP treatment (Fig. 3*C*); APEH is a good example. In contrast to the complete overlap in tryptic and nontryptic peptides observed for LDHA, there was no overlap of tryptic and nontryptic sequences for APEH. Figure 4*D* highlights the change in intensity for a selected APEH tryptic peptide (enclosed by *box* in Fig. 4*C*) and an adjacent nontryptic sequence. Here, the addition of DFP led to a reduction in the intensity of the tryptic peptide (30% reduction with DFP, *p* = 0.172), while still markedly suppressing the detection of the nontryptic sequence. The summed results for these two types of DFP effects are shown in Figure 4*E*. There is a twofold increase (*p* = 0.017) in tryptic peptide intensity for LDHA in the presence of DFP and a 34% decrease (*p* = 0.097) in tryptic peptide intensity for APEH, corresponding with the observed decrease in protein abundance. We identified additional examples of proteins that were consistently increased in DFP-treated samples and found a similar trend of overlapping tryptic and nontryptic sequences (Supplemental Fig. S3).

For those proteins with decreased abundances with DFP, we observed both increased and decreased relative intensities for individual tryptic peptides, and the patterns were protein specific; the net effect was a deceased protein abundance from summed tryptic peptide intensity. In contrast to proteins that increase in apparent abundance with DFP, the coverage maps for these decreasing proteins do not have canonical rules, with some patterns resembling APEH (*e.g.*, ELANE and CTCF, Supplemental Fig. S4, *A* and *B*), and others showing a more complete overlap between tryptic and nontryptic sequences (*e.g.*, RPL17 and RDP9, Supplemental Fig. S4, *C* and *D*). Although treatment with DFP still acts to decrease generation of nontryptic peptides, the mechanisms responsible for the decrease in tryptic signals with DFP treatment are not yet clear.

We next investigated whether we could correct for the loss of tryptic signal in the absence of DFP computationally by using both tryptic and nontryptic peptide intensities. Overall, we observed a similar pattern of clustering according to case and DFP treatment (Fig. 3*A*, Supplemental Fig. S5*A*). Considering the combined tryptic and nontryptic peptide signals, we found a negative correlation between DFP-treated and DFP-untreated samples (Supplemental Fig. S5, panel *B*), similar to that observed when considering only the tryptic signal (Fig. 3*B*). Furthermore, a similar number of proteins increased and decreased with DFP treatment for case 1 (tryptic only—254 proteins decreased, 345 increased, Figure 3*C*; tryptic plus nontryptic—293 proteins decreased, 310 increased, Supplemental Fig. S5*C*). Of the 10 proteins consistently changed across AML cases and selected for detailed peptide analysis (Fig. 4 and Supplemental Figs S3 and S4), four of the five proteins remained significantly decreased with DFP treatment when both tryptic and nontryptic signal was considered. Of the five proteins found to be consistently increased when considering only tryptic peptides, one remained increased and one showed a decreased abundance when the combined tryptic and nontryptic signal was used. In addition, the numbers of consistently increased and decreased proteins were similar whether considering only tryptic peptides (Fig. 3*D*) or the combined tryptic and nontryptic signal (Supplemental Fig. S5*D*). From these results (Fig. 3 and Supplemental Fig. S5), we concluded that the alteration in tryptic signals because of endogenous serine protease activity could not be corrected by using the combined tryptic and nontryptic signal.

### Myeloid Serine Proteases are Present and Active in Some Solid Tumors With Inflammatory Infiltrates

Inflammatory cells often infiltrate solid tumors, including pancreatic cancer (13), squamous cell lung carcinoma (14), and inflammatory breast cancer (20). We therefore investigated whether myeloid serine protease activity could be detected in existing large-scale CPTAC datasets from these tumor types.

**Fig. 5:**
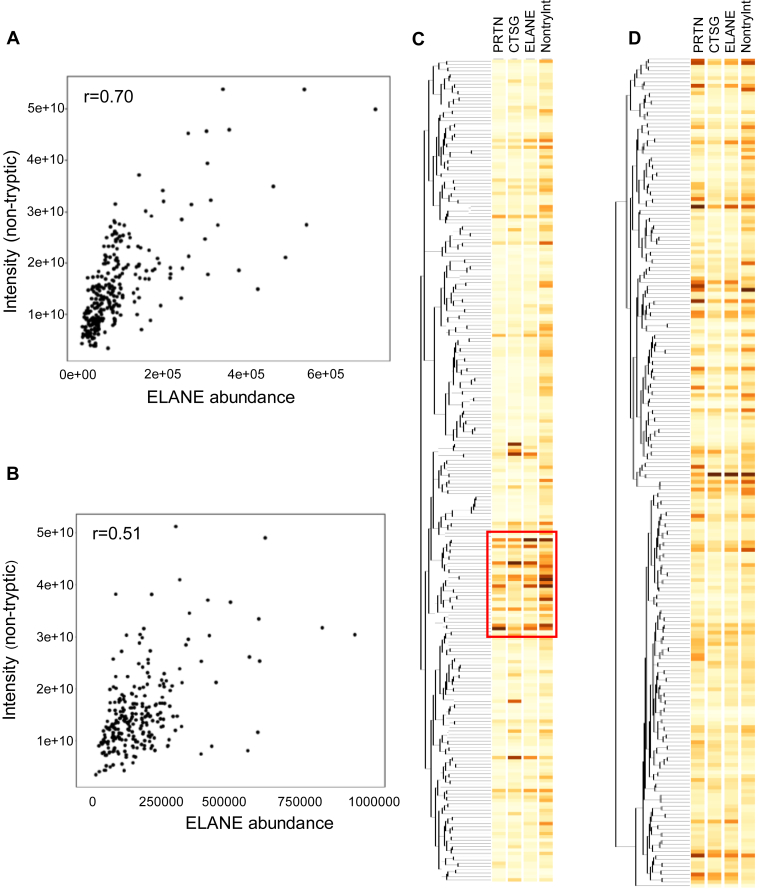
**Correlation of nontryptic peptide intensity with ELANE abundance in large-scale studies of pancreatic and lung cancer.***A*, Pearson's correlation of ELANE abundance and peptide intensities generated from nontryptic cleavages in a large-scale study of pancreatic cancer (13). *B*, Pearson's correlation of ELANE abundance and peptide intensity from nontryptic cleavages in a large-scale study of squamous cell lung carcinoma (14). *C*, unsupervised hierarchical cluster analysis of pancreatic cancer proteins and patients. *Box* indicates samples associated with higher ELANE abundance that also clustered near each other in unsupervised clustering. *D*, unsupervised hierarchical cluster analysis of squamous cell lung carcinoma proteins and patients. ELANE, neutrophil elastase.

We compared the relative abundance of ELANE with the summed intensity of nontryptic peptides detected in these datasets, allowing for any residue at the C terminus except arginine or lysine, and considering nontryptic residues at the preceding N-terminal residue in the protein sequence. Figure 5, *A* and *B* shows the correlation between ELANE abundance and nontryptic peptide intensity for pancreatic and lung cancer datasets, respectively. This analysis demonstrates a strong positive correlation (*r* = 0.70) between ELANE abundance and nontryptic peptide intensity in the pancreatic cancer dataset (Fig. 5*A*) and to a somewhat lesser extent in squamous cell lung cancer (*r* = 0.51, Fig. 5*B*). Both have evidence for nontryptic cleavage activity in many of these specimens.

We also determined whether the presence of ELANE activity might be apparent in the unsupervised hierarchical clustering of proteins from these studies. Interestingly, the high ELANE expressers tended to group together in the unsupervised cluster analysis of pancreatic cancer proteins identified with tryptic peptides (Fig. 5*C*, highlighted with *box*). Further studies will be required to determine whether this grouping of cases is due to the underlying biology of these tumors or due to alterations in protein abundance measurements caused by ELANE activity. In contrast, no apparent clustering of high ELANE expressers was observed in the unsupervised hierarchical cluster analysis of the lung cancer specimens (Fig. 5*D*).

**Fig. 6:**
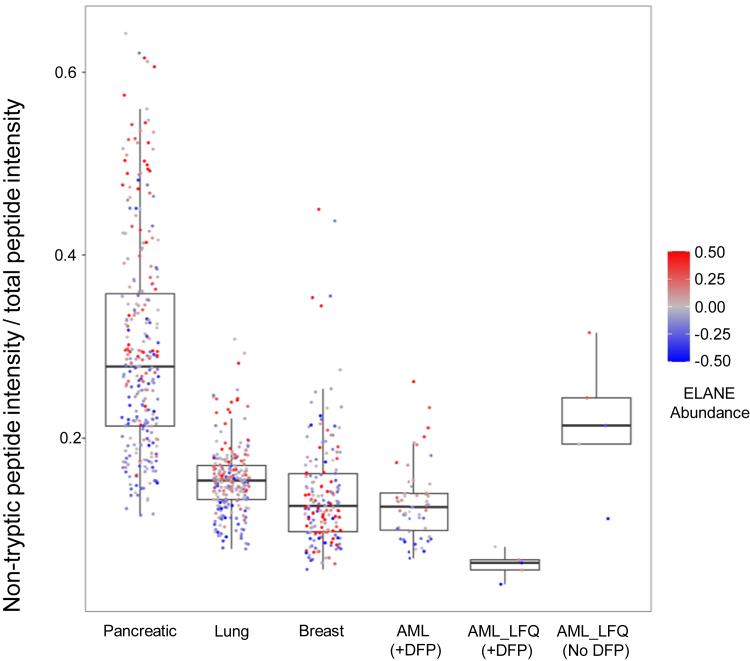
**Intensity proportion of nontryptic peptides in four cancer types.** Each point represents a patient in each of the indicated studies (pancreatic: (13); lung: (14); breast: (20), 2020; AML [+DFP]: (15); AML_LFQ: present study). *Y*-axis indicates the percentage of total intensity detected in that sample, which was assigned to nontryptic peptides. Samples are color-coded by ELANE intensity with respect to the group means of each box–whisker condition. AML, acute myeloid leukemia; DFP, diisopropyl fluorophosphates; ELANE, neutrophil elastase; LFQ, label-free quantitation.

We also examined the relative intensity of nontryptic peptides in four cancer types from CPTAC datasets (Fig. 6). Greater proportions of nontryptic signals were observed with the pancreatic cancer samples, where samples with greater nontryptic proportions were enriched for higher ELANE intensities. The intensity ratio of nontryptics to total peptide intensity is similar to that observed for AML (34%) without DFP treatment. Within AML cases, a drastic reduction in nontryptic intensity was observed for samples with DFP treatment.

To better understand whether the myeloid serine proteases are responsible for these alterations in solid tumor proteomic datasets, we determined whether the nontryptic peptides observed demonstrated cleavage specificity associated with ELANE activity (cleavage C terminal to A, I, or V). We saw a strong correlation between ELANE abundance and the abundance of nontryptic peptides expected to be generated by ELANE–PRTN3 in the pancreatic cancer dataset (Supplemental Fig. S6). This suggests that ELANE (and possibly PRTN3, with the same specificity) is responsible for the generation of these nontryptic peptides. The ELANE–PRTN3 specificity was not as apparent in the lung cancer dataset, whereas the breast cancer dataset showed an anticorrelation between ELANE expression and intensity of nontryptic peptides cleaved at A, I, and V residues (the number of cases in that sample set with inflammatory breast cancer was not stated).

## Discussion

Previous studies have suggested that enhanced inhibition of endogenous proteases is needed to identify specific proteins in myeloid cells (5, 6). However, proteomic studies of myeloid cells to assess the requirement for inhibition of the highly abundant and highly active azurophil granule serine proteases (ELANE, PRTN3, CTSG, and NSP4), produced primarily at the promyelocyte/promonocyte stage of differentiation, have not been reported.

We demonstrate here that inhibition of the granule serine proteases with a membrane-permeable serine protease inhibitor (DFP) (7, 8) prior to cell lysis significantly alters protein abundance measurements assessed by tryptic digestion and MS. These studies demonstrated an increase in the number of proteins identified by 9 to 31% from five AML cases, using LFQ. In all cases, DFP treatment increased the number of tryptic peptides and decreased the number of nontryptic peptides detected and/or their intensities.

Using a standard high-precision proteomic method for isobaric peptide labeling (2), we investigated the effect of DFP on the global quantitation of proteins from three AML cases with high ELANE expression. The measured abundance of ∼20% of the proteome was significantly affected by pretreatment of AML samples with DFP: about half were increased. For these proteins, all tryptic peptides for individual proteins were significantly increased in intensity. Many of the nontryptic cleavage sites occurred within tryptic peptides, suggesting that inhibition with a serine protease inhibitor has a “tryptic sparing” effect, resulting in an increase in measured protein relative abundance. Surprisingly, about half of the proteins with DFP-related abundance changes were decreased with DFP treatment. The sequence coverage maps for these decreasing proteins did not show a clear tryptic-sparing effect; for these proteins, we suspect that DFP treatment may inhibit a protease(s) that actually enhances tryptic peptide generation, perhaps by making some tryptic cleavage sites more accessible.

The detection of nontryptic peptides yielded important information on the specificity of endogenous proteases in the AML samples. We determined that the endoproteolytic cleavages at amino acids other than Lys and Arg (specific for trypsin) were usually at Ala, Iso, and Val amino acid residues (and not at Leu residues), corresponding to the specificity of ELANE and PRTN3 (28). These findings suggest that analysis of nontryptic peptides can be used to infer the activity of myeloid proteases from any tissue type, since these proteases are expressed exclusively in myelomonocytic cells.

Inflammatory cells often infiltrate solid tumors, including pancreatic cancer (13), squamous cell lung carcinoma (14), and inflammatory breast cancer (20), and myeloid infiltration is thought to alter the tumor microenvironment in ways that can be either beneficial or deleterious to tumor cells (10, 11, 12). The work in this study suggests that it may be possible to use proteomic measurements from bulk samples to assess the degree of myeloid infiltration in a tumor, through either the detection of neutrophil granule proteases (*e.g.*, ELANE–PRTN3) directly or the detection of nontryptic peptides generated by these active myeloid serine proteases. The effects of this myeloid infiltration on tumor biology may be an area of future research.

Our results suggest novel quality control methods to assess the presence of active endogenous proteases during sample processing. The current CPTAC protocol includes an optional quality control step involving single-shot LC–MS analysis prior to TMT labeling (2). If these single shot data are available and searches are performed that allow for nonspecific enzyme cleavages, an assessment of the activity of the endogenous proteases can be inferred, and optimization of endogenous protease inhibition can be pursued. Our results also show that if DFP is used, active site serine modifications may be indicative of adequate inhibition. This approach of targeted searching for active site modifications may be applicable for the optimization of protease inhibitors more generally.

We also explored the possibility that datasets could be reanalyzed by including the nontryptic peptide signal; however, we found that a large number of proteins remained both increased and decreased in the absence of adequate protease inhibition. Thus, it appears that proteomic data generated in the absence of adequate protease inhibition are not readily correctable by inclusion of nontryptic signals, although future efforts in this direction would certainly be welcomed.

In summary, the pretreatment of cryopreserved AML samples with DFP has a striking and reproducible effect on the measurement of tryptic peptides with MS. These data show that the standard approach of endogenous protease inhibition does not completely inactivate ELANE, PRTN3, and CTSG. Finally, our studies suggest an approach to monitor and optimize the inhibition of endogenous serine proteases in proteomic studies.

## Data Availability

The MS proteomics data are available *via* ProteomeXchange with identifier PXD044112.

## Supplemental data

This article contains supplemental data.

## Conflict of interest

The authors declare no competing interests.
