# Supplementary material for: Stabilizing the Proteomes of Acute Myeloid Leukemia Cells: Implications for Cancer Proteomics
Source: Mol Cell Proteomics. 2024 Jan 12;23(2):100716. doi: 10.1016/j.mcpro.2024.100716 (PMC10864662; doi:10.1016/j.mcpro.2024.100716)
Supplement: Supplemental Data [file mmc1.pdf]

Stabilizing the Proteomes of Acute Myeloid Leukemia Cells: Implications for Cancer Proteomics

Robert Sprung, Qiang Zhang, Michael H. Kramer, Matthew C. Christopher, Petra Erdmann-Gilmore, Yiling Mi, James P. Malone, Timothy J. Ley, R Reid Townsend

Supplemental Materials

Figure S1	Nontryptic cleavage specificity in the absence of DFP
Figure S2	Proteomic analysis of an AML case with high expression of ELANE in the presence or absence of DFP
Figure S3	Sequence coverage of proteins increased with DFP treatment from case 1
Figure S4	Sequence coverage of proteins decreased with DFP treatment from case 1
Figure S5	Pearson Correlations of ELANE abundance and non-tryptic peptide intensities in large scale tumor and Leukemia studies
Table S1	AML Case descriptors and LC-MS data files
Table S2	All Peptides by Case -LFQ
Table S3	Identification of tryptic and non-tryptic peptides from five AML cases with high and low expression of ELANE
Table S4	Number of proteins identified by LFQ proteomics with a minimum of 2 tryptic peptides
Table S5	DFP Adduct Database Search Tryptic Peptides
Table S6	Protein quantification from TMT 11-plex tryptic peptides with and without DFP
Table S7	Tryptic peptides used for protein quantification from TMT 11-plex with and without DFP
Table S8	Changes in TMT relative abund. with DFP treatment
Table S9	Protein quantification from LFQ tryptic peptides with and without DFP
Table S10	Proteins with significant change in abundance with DFP treatment using Label-Free Quantitation

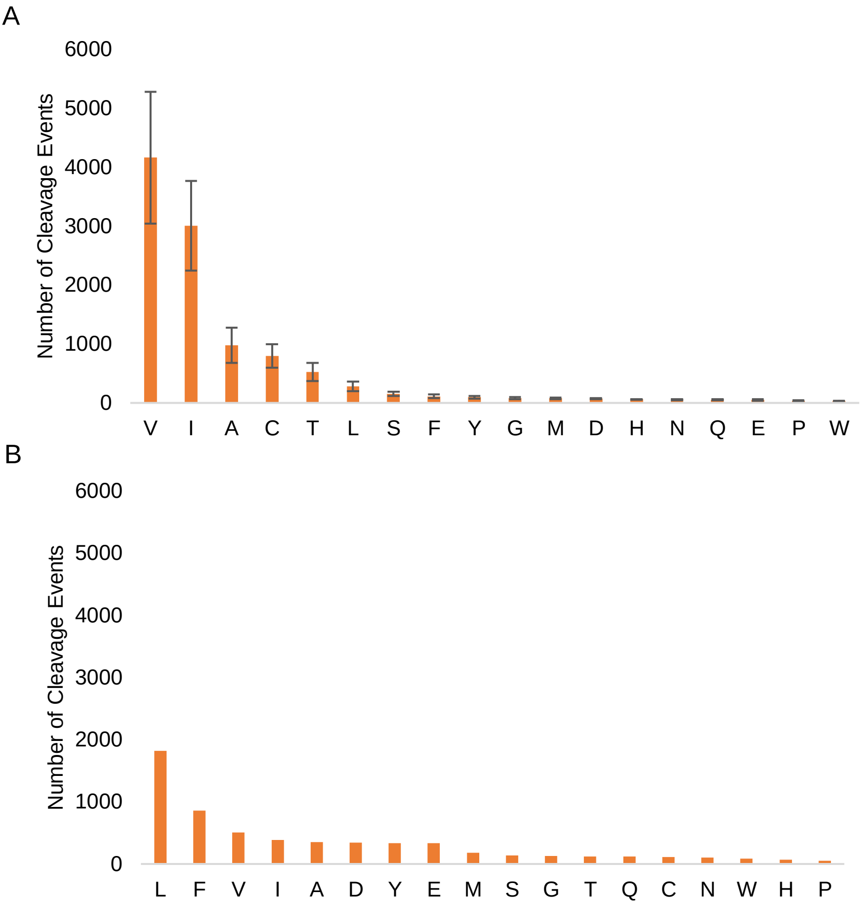

Supplemental Figure S1. Nontryptic cleavage specificity in the absence of DFP. Both C-terminal and flanking N-terminal residues were considered. *A,* Case 1. Error bars represent standard deviation of the process replicates. *B*, Case 5.

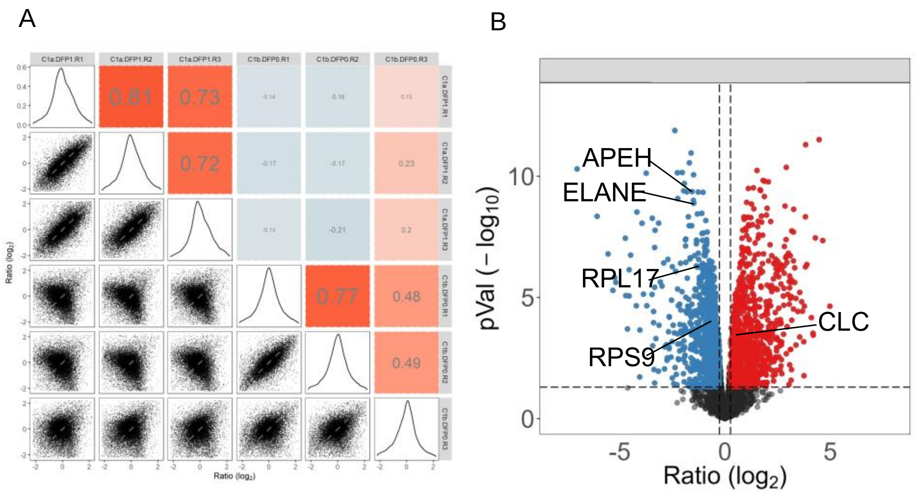

Supplemental Figure S2. Proteomic analysis (unlabeled tryptic peptides) of an AML case with high expression of ELANE in the presence or absence of DFP. *A*, Pairwise correlations of the peptide relative abundances for the 6 replicates of case 1, 3 in the presence and 3 in the absence of DFP. *B*, Relative fold change and P values generated from digestion replicate determinations of protein abundance for AML Case 1, comparing those treated with DFP to those without DFP. Increased abundance indicates more protein measured in the replicates treated with DFP.

A GAPDH

MGKVKVGVNGFGRIGRLVTRAAFNSGKVDIVAINDPFIDLNYMVYMFQYDSTHGKF
HGTVKAENGKLVINGNPITIFQERDPSKIKWGDAGAEYVVESTGVFTTMEKAGAHLQ
GGAKRVIISAPSADAPMFVMGVNHEKYDNSLKIISNASCTTNCLAPLAKVIHDNFGIV
EGLMTTVHAITATQKTVDGPSGKLWRDGRGALQNIIPASTGAAKAVGKVIPELNGK
LTGMAFRVPTANVSVVDLTCRLEKPAKYDDIKKVVKQASEGPLKGILGYTEHQVVS
SDFNSDTHSSTFDAGAGIALNDHFVKLISWYDNEFGYSNRVVDLMAHMASKE

B TUBB

MREIVHIQAGQCGNQIGAKFWEVISDEHGIDPTGTYHGDSDLQLDRISVYYNEATGG
KYVPRAILVDLEPGTMDSVRSGPFGQIFRPDNFVFGQSGAGNNWAKGHYTEGAEL
VDSVLDVVRKEAESCDCLQGFQLTHSLGGGTGSGMGTLLISKIREEYPDRIMNTFS
VVPSPKVSDTVVEPYNATLSVHQLVENTDETYCIDNEALYDICFRTLKLTTPTYGDL
NHLVSATMSGVTTCLRFPGQLNADLRKLAVNMVPFPRLHFFMPGFAPLTSRGSQQ
YRALTVPELTQQVFDAKNMMAACDPRHGRYLTVAAVFRGRMSMKEVDEQMLNVQ
NKNSSYFVEWIPNNVKTAVCDIPPRGLKMAVTFIGNSTAIQELFKRISEQFTAMFRR
KAFLHWYTGEGMDEMEFTEAESNMNDLVS

C HNRNPK

METEQPEETFPNTETNGEFGKRPAEDMEEEQAFKRSRNTDEMVELRILLQSKNAG
AVIGKGGKNIKALRTDYNASVSVPDSSGPERILSISADIETIGEILKKIIPTLEEGLQLP
SPTATSQLPLESDAVECLNYQHYKGSDFDCELRLLIHQSLAGGIIGVKGAKIKELRE
NTQTTIKLFQECCPHSTDRVVLIGGKPDRVVECIKIILDLISESPIKGRAQPYDPNFYD
ETYDYGGFTMMFDDRRGRPVGFPMRGRGGFDRMPPGRGGRPMPPSRRDYDDM
SPRRGPPPPPPGRGGRGGSRARNLPLPPPPPPRGGDLMAYDRRGRPGDRYDGM
VGFSADETWDSAIDTWSPSEWQMAYEPQGGSGYDYSYAGGRGSYGDLGGPIITT
QVTIPKDLAGSIIGKGGQRIKQIRHESGASIKIDEPLEGSEDRIITITGTQDQIQNAQYL
LQNSVKQYSGKFF

D CLC

MSLLPVPYTEAASLSTGSTVTIKGRPLACFLNEPYLQVDFHTEMKEESDIVFHFQVC
FGRRVVMNSREYGAWKQQVESKNMPFQDGQEFELSISVLPDKYQVMVNGQSSYT
FDHRIKPEAVKMVQVWRDISLTKFNVSYLKR

Supplemental Figure S3. Sequence coverage of proteins increased with DFP treatment from case 1. *A*, GAPDH. *B*, TUBB. *C*, HNRNPK. *D*, CLC. Tryptic peptides are shown in red, non-tryptic peptides in blue, and peptide sequences common to both appear in purple.

A ELANE

MTLGRRLACLFLACVLPALLLGGTALA**SEIVGGRRARPHAWPFMVSLQLRGGHFCGAT**
LIAPNFVMSAAHCVANVNVRAV**RVVLGAHNLSRREPTRQVFAVQRIFENGYDPVNLLN**
DIVILQLNGSAT**INANVQVAQLPAQGRRLGNGVQCLAMGWGLLGR**NRGIASVLQELNV
TVVTSLCR**RSNVTLVRGRQAGVCFGDSGSPLVCNGLIHGIASFVRGGCASGLYPDAFA**
PVAQFVNWIDSIIQRSEDNPCPHPRDPDPASRTH

B CTCF

MEGDAVEAIVEESETFIKGKERKTYQRRREGGQEEDACHLPQNQTDGGEVVQDVNSS
VQMVMMEQLDPTLLQMKTEVMEGTVAPEAEAAVDDTQII**TLQVVNMEEQPINIGELQLV**
QVPVPVTVPVATTSVEELQGAYENEVSKEGLAESEPMICHTLPLPEGFQVVKVGANG
EVETLEQGELPPQEDPSWQK**DPDYQPPAK**KTKKTKKSK**LRYTEEGKDVDVSVYDFEE**
EQQEGLLSEVNAEKVVGNMKPPKPTKIKKKGVKKTFQCELCSYTCPRRSNLDRHMKS
HTDERPHKCHLCGRAFRTVTLLR**NHLNTHTGTRPHKCPDCDMAFVTSGELVR**HRRYK
HTHEKPFK**CSMCDYASVEVSK**LKRHIR**SHTGERPFQCSLCSYASR**DTYKLKRHMRTHS
GEK**PYECYICHAR**FTQSGTMKMHILQKHTENVAK**FHCPHCDTVIAR**KSDLGVHLRK**QH**
SYIEQGKKCR**YCDAVFHERYALIQHQK**SHKNEKRFK**CDQCDYACR**QERHMIMHKRTH
TGEK**PYACSHCDK**TFRQK**QLLDMHFKRYHDPNFVPAAFVCSK**CGKTFTRRNTMAR**H**
ADNCAGPDGVEGENGGETKKSKRGRKRKMRSKKEDSSDSENAEPDLDDNEDEEEPA
VEIEPEPEPQPVTPAPPPAKKRRGRPPGRTNQPKQNQPTAIIQVEDQNTGAIENIIVEVK
KEPDAEPAEGEEEEAQPAATDAPNGDLTPEMILSMMDR

C RPL17

M**VRYSLDPENPTK**SCKSRGSNLRVHFKNTRETAQAIKGMHIRKATKYLKDVTLQKQCV
PFR**RYNGGVGR**CAQAK**QWGWTQGR**WPKK**SAEFLLHMLKNAESNAELKGLDVDSLVI**
EHIQVNKAPKMRRRTYRAHGR**INPYMSSPCHIEMILTEKEQIVPKPEEEVAQKK**KISQK
KLKKQKLMARE

D RDP9

MPVARSWVCRKTYVTPRRPFEK**SRLDQELKLIGEYGLR**NKREVWRVKFTLAKIRKAAR
ELLTLDEKDPR**RLFEGNALLRR**LV**RIGVLDEGKMKLDYILGLKIEDFLERRLQTQVFK**L
GLAKSIHHARVLIRQRHIRVRK**QVVNIPSFIVR**LDSQK**HIDFSLRSPYGGGRPGR**VKRKN
AK**KGQGGAGAGDDEEED**

Supplemental Figure S4. Sequence coverage of proteins decreased with DFP treatment from case 1. *A*, ELANE. *B*, CTCF. *C*, RPL17. *D*, RDP9. Tryptic peptides are shown in red, non-tryptic peptides in blue, and peptide sequences common to both appear in purple.

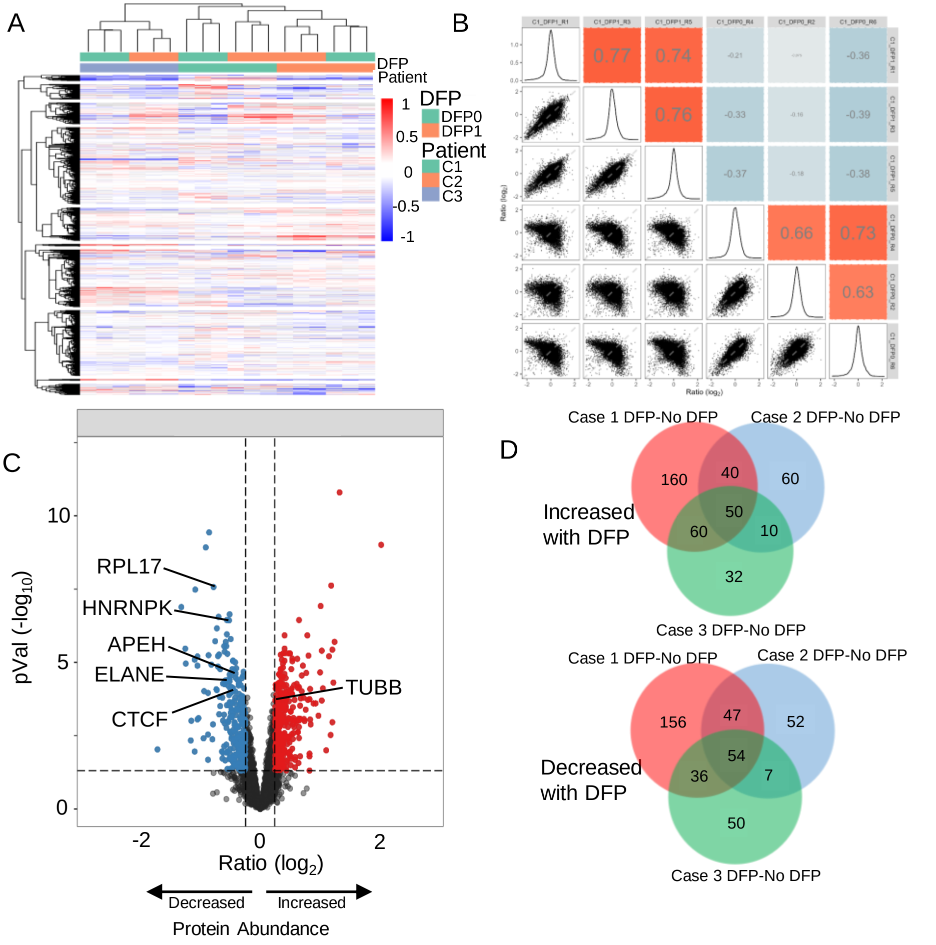

Supplemental Figure S5 Proteomic analysis (TMT-labeled tryptic + non-tryptic peptides) of AML cases with high expression of ELANE in the presence or absence of DFP. *A*, Unsupervised protein hierarchical cluster analysis for 3 cases, each with 6 LysC/trypsin digest replicates (3 +DFP and 3 no DFP). Supplemental table 5 contains a complete listing of proteins represented in this analysis. *B*, Pairwise correlations of the peptide relative abundances for the 6 replicates of case 1, 3 in the presence and 3 in the absence of DFP. *C*, Relative fold change and P values generated from digestion replicate determinations of protein abundance for AML Case 1, comparing those treated with DFP to those without DFP. Increased abundance indicates more protein measured in the replicates treated with DFP. *D*, Boolean analysis of proteins that changed significantly ($P < 0.05$) with DFP treatment in each of the three cases. Proteins increasing with DFP treatment are shown in the upper Venn diagram, those decreasing with DFP treatment are shown in the lower Venn diagram. A selection of the shared proteins is shown in panel C.

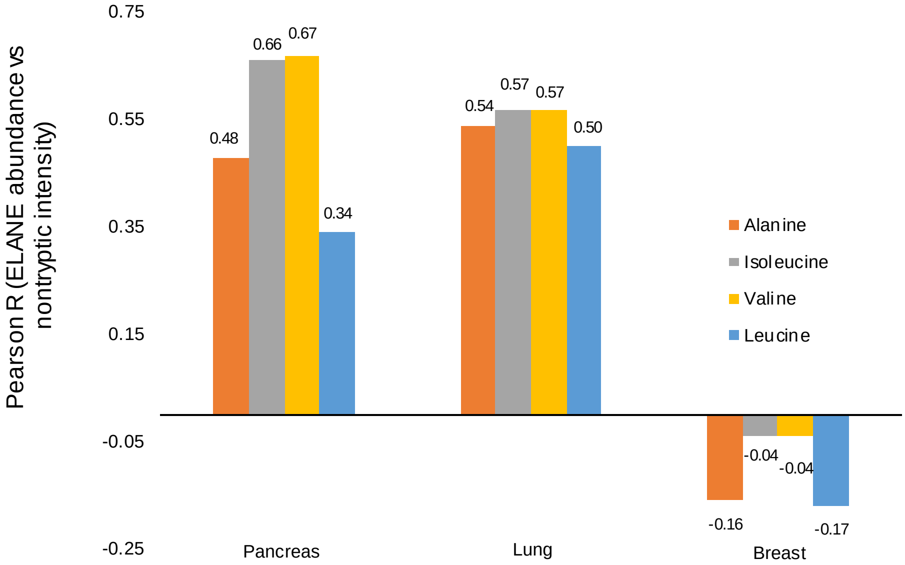

Supplemental Fig. S6 Pearson Correlations of ELANE abundance and non-tryptic peptide intensities in large scale tumor and Leukemia studies. Pearson correlations of the total identified non-tryptics are shown for pancreatic [13], squamous cell lung carcinoma [14], breast [20] cancers. Pearson correlations for peptides with N-terminal flanking and C-terminal residues Alanine, Isoleucine, Valine, Leucine, and the total from nontryptic peptides are shown as individual bars.
